# Integrated Place-Based Primary Interventions: Levers and Tensions Related to Multilevel Governance for Community Integrated Pathways, A Multiple Case Study

**DOI:** 10.1177/11786329241234997

**Published:** 2024-03-11

**Authors:** Anna Goudet, Chantal Doré, Shelley-Rose Hyppolite, Nancy Lévesque, Jean-Alex Joseph, Danielle Maltais, Denis Bourque, Lara Maillet

**Affiliations:** 1 National School of Public Administration, Montreal, QC, Canada; 2 Université de Sherbrooke, Institut Universitaire de première ligne en santé et services sociaux, Sherbrooke, QC, Canada; 3 Université Laval, Centre intégré universitaire de santé et des services sociaux de la Capitale-Nationale, Quebec, QC, Canada; 4 National School of Public Administration, QC, Canada; 5 Université de Sherbrooke, Sherbrooke, QC, Canada; 6 Université du Québec à Chicoutimi, Chicoutimi, QC, Canada; 7 Université du Québec en Outaouais, Gatineau, QC, Canada; 8 National School of Public Administration, Chaire de recherche du Canada en système adaptatifs en santé et services sociaux, Institut Universitaire de première ligne en santé et services sociaux (IUPLSSS), Montreal, QC, Canada

**Keywords:** Community-integrated intervention, care and services pathway, governance, health system, vulnerability, case study

## Abstract

Integrated Place-Based Primary Interventions (IPPIs) are considered an innovative response to the challenges and complex issues faced in disadvantaged areas where traditional institutional services have difficulty reaching people in vulnerable situations. IPPIs are an innovative approach to the delivery of in services, conceived as an original community-based local care and service pathways. However, these intervention practices require adaptive modes of governance. In this article, we explore how and to what extent the mode of governance of IPPIs influences the performance of community-integrated pathways. To this end, using a qualitative exploratory multiple-case study design (observation and semi-structured interviews), we describe 4 IPPIs in 3 territories in Quebec. This includes an examination of the levers of action and tensions related to their governance and the performance levels of the community-integrated pathways. We conclude that collaborative and shared multilevel governance, despite its demanding nature, appears to contribute to the longevity of the actions and benefits of IPPIs and could prevent their relevance from being questioned.

## Introduction

The implementation of integrated place-based primary interventions (IPPIs) rooted in the living environments of citizens is considered to be an innovative response to the complex challenges and issues faced in disadvantaged areas which institutional services have difficulty in reaching people in vulnerable situations. The need has become even more pronounced in Quebec, Canada, since the 2015 reform1 that led to the creation of regional megastructures resulting from the amalgamation of local and regional establishments (Health and Social Services Centers, Hospital Center, Long-term Care Facilities, etc.) into a single establishment: Integrated and University affiliated Health and Social Services Establishment (HSSE). This reform has also given an important place to accountability in public management, and has engendered an increase in the compartmentalization of social interventions.^1-4^ IPPIs in health and social services represent an alternative way to offer such services, which are decentralized in living environments frequented by citizens that are characterized by situations of social vulnerability. For example, one of the IPPIs studied is directly based in an apartment in a rental building within the targeted neighborhood. There, workers approach individuals who would not generally approach them, building trust with the citizens and collaborating with local partners, to better respond to the individual and collective needs of the communities. For example, IPPI workers will take part in community social and festive events, participate in food distributions and, in short, be part of neighborhood life. The effects of the IPPIs on individuals and communities have been recognized and evaluated.^5-12^

IPPIs constitute an innovation in the provision of institutional services that can be considered as an original method for the provision of community-integrated care and service pathways. The mode of organization for social and medical services “through pathways” has been on the agenda in Quebec since 2015. Originally developed in Anglo-Saxon countries and generally centered around care episodes, the objective of the pathway approach is to formalize the sequencing of care provision and interventions by multidisciplinary teams, according to robust data and supported by good practice guides. In Quebec, there is a broader notion of pathways, which includes sanitary and social dimensions, is centered around the user and is conceived of as several overlapping care and service episodes over long periods, from an intersectoral perspective and within a targeted territory.^13,14^

Nonetheless, to our knowledge, no research has examined the tensions that may exist between these innovative intervention practices and the management modalities that underpin and support them. Even more broadly, scientific research into cross-sectoral and cross-organizational initiatives generally overlooks governance structures and processes; this is particularly the case in the fight against homelessness in Quebec and internationally.^15^ Yet, during a study on accountability provisions for 4 IPPIs in Quebec,^8^ governance emerged as a central issue, particularly the types of governance and the context in which they are integrated. This article considers the following question: to what extent does the type of governance of the IPPIs influence their performance in terms of community-integrated pathways? The design of our multi-case qualitative study enables us to highlight the specific and common characteristics of each of the 4 cases, and thus to bring out the internal and environmental factors linked to the governance processes that contribute to the performance and sustainability of these IPPIs.

We will begin by delineating our conceptual framework and methodological approach, then we will briefly describe each of the 4 cases before discussing the governance issues and their potential effects on the performance of community-integrated pathways.

## Conceptual Framework

The initial research project focused on the accountability of IPPIs. The aim was to grasp the points of tension in current ways of doing things, and to develop accountability tools more appropriate to this type of intervention. The data collected highlighted a central issue: IPPI governance within the health and social services establishment and with external partners. Indeed, to meet the needs of populations within targeted territories, IPPIs must always adapt, and the governance in which IPPIs are integrated have a major influence on their ability to do so. To analyze these issues, we have developed a conceptual framework that includes the initial conditions for IPPI implementation, the levers of action for governance and the performance dimensions of community-based care and service trajectories.

### The community-based intervention: Definition and initial conditions

Cross-sector collaboration is at the heart of IPPI, along with 5 specific features.^16,17^ (1) This intervention develops over the course of time. Its results and effects are evaluated in the short-, medium-, and long-terms. (2) The IPPI is local both in the spatial and relational senses: it takes place close to populations and is visible, accessible, available and present in living environments. The IPPI workers aim to build trust with the community. (3) It allows the integration of health and social services through collaboration between the different services offered within HSSEs, in which collaboration with partners is prioritized. (4) The IPPI territory is both a geographical territory—often restricted to a micro-territory—as well as a “lived” and “perceived” territory, which relates to experiences and impressions.^18^ (5) The IPPI is innovative in nature and gives rise to new arrangements at the organizational level and in daily practices. Innovation is based on the “transformative and systemic scope”^19^ of the IPPI, including its influence on the social determinants of health, which can contribute toward reducing social health inequalities.

This mode of intervention appears as a response to the population responsibility set forth in the Act respecting health and social services and in Quebec’s 2015 to 2025 national public health program,^20,21^ which requires the development of new working methods in health and social services establishments (HSSEs) and their territorial networks.

To understand how this intersectoral collaboration works, and its results in terms of performance, it is important to start by understanding how it came about, and where it starts from. To this end, we draw on the framework of Bryson et al,^22^ who propose an analytical reading of the initial conditions of intervention implementation. Indeed, this framework, which is based on an extensive selection of scientific texts, is particularly useful for understanding the dynamics at work within intersectoral collaborations, both internally and within their environments. The initial conditions for intersectoral collaboration notably refer to environmental factors (including a certain degree of “turbulence”) and the background of the collaboration (including the notion of sector failure as a precondition for collaboration, and other specific and immediate preconditions affecting the formation of collaborations). According to Bryson et al,^22^ the initial conditions for implementing an initiative can influence not only the intervention itself, but also the formation and adaptation of governance. We can therefore expect that the initial conditions under which the IPPIs emerge will also influence the formation and adaptation of their mode of governance.

### Governance in the context of health and social services establishments

Governance refers “to the organizational design of the care system and to the sharing of responsibilities and ability to influence among the different entities that constitute it, to the systems and mechanisms of production and dissemination of information and to the financing modalities of the organizations and professionals” (p. 3).^23^

Our conceptual framework for multilevel governance is based principally on the work of Folke et al.,^24^ Lamothe,^25^ and Maillet et al.^26^ Multilevel governance is shaped at 3 levels (operational, tactical, strategic): the operational corresponds to the ground clinical-administrative level, the tactical to the management level, and the strategic to the “hierarchical” decision-making level.^27,28^ From this perspective, governance becomes a space in which the voice of actors at the operational level benefit from the credibility associated with the tactical and strategic levels. Conversely, the administrators of the strategic level have an ongoing need for legitimacy from the actors at the other levels, notably from the health professionals. The tactical level is responsible for translating strategic guidelines to the operational level and transferring the actions undertaken at the operational level to the strategic level.^29^ In this way, coherence is created between the 3 levels, which allows the process of adaptation and organization to be strengthened and to be explicitly linked to the concept of equity within the populations being served, notably the most vulnerable.^30,31^

By using action levers the governance structures can ensure the convergence of heterogeneous actions based on collective interests toward a collective process that is more homogeneous and shares a common vision.^32^ Thus, we seek to identify the levers of multi-level governance that foster adaptation of the healthcare organization, to implement performing IPPIs that meet the emerging needs of the populations served.

The data collected have guided the analysis on the levers related to structure, resources, communications, connectivity and knowledge (adapted from Maillet et al^32^) (Table 1).

**Table 1.** Governance levers and definitions.

Action lever	Definition
Structure	Roles and functions of actors who are officially defined by the organization in the implementation of the IPPI. These levers refer in particular to the leadership of the project leaders, the alignment of the vision of the IPPI’s orientation between the strategic, tactical and operational levels (or competing institutional logics), the power relationships and the decision-making modalities (horizontal or vertical). These structural levers ultimately reflect the strategic support granted (or not) to the IPPI.
Resources	Four types of resources: the human, financial, material and time resources allocated for the implementation of the IPPI. They translate the priority given to the IPPI.
Communication	Information mechanisms established to collect and keep all the actors invested in IPPI well informed, both within and outside of the organization (including the modalities for accountability).
Connectivity	Formal or informal links established in time and space between the actors within an organization and/or with external partners.
Knowledge	Expertise, learning and knowledge of the different actors regarding the philosophy of the IPPI and of the relevant territory.

### The performance of the pathways

We conceive the performance of care and service pathways to be dynamic and multidimensional.^13,14,33^ Therefore, the analysis and measurement of performance requires simultaneous study of the different dimensions that constitute it as well as the nature of the relationships between these dimensions. It is also necessary to contextualize the performance based on the characteristics of the population and territory being targeted by the community-integrated pathway, as well as the partners involved. In other words, it is necessary to understand the needs of the target population and the collective and community resources available to comprehend the performance of a pathway.

Based on the mandates of the IPPIs studied, we are targeting certain dimensions drawn from the Performance analysis model (PAM) for pathways:^14^ accessibility, adjustment to the needs of the population, coordination and continuity, equity (Table 2).

**Table 2.** Performance dimensions and Definitions.

Dimensions	Definitions
Accessibility of care and services	Level of ease for a citizen-user to obtain care or services that meet their needs through the reduction of geographical, economic, individual, relational, sociocultural and organizational barriers.
Adjustment to the needs of the population	Ability of a pathway to adapt to respond to the needs of the target population based on the context, through collaboration between partners.
Coordination and continuity of care and services	Ability to involve the actors (including the external partners) and the care and services in a synchronized manner, to ensure streamlined responses to the needs of the citizens-users, according to a time sequence involving several resources.
Equity	Absence of any unjust and avoidable gaps in the areas of health care and social services. Equity requires an adjustment in terms of the health care and social services offered based on the social disadvantages of citizens-users in achieving similar health and well-being results across different population groups. This dimension is both at the heart of the philosophy of the IPPIs and of the PAM.

## Methodology: A Multiple Case Study

Using an exploratory multiple case study approach,^34^ the research focus on 4 IPPIs linked to 3 HSSEs in 3 regions in Quebec, Canada. The qualitative multiple-case study design enables a pragmatic, precise and in-depth analysis of the particularities, complexity and comprehensiveness of the cases^34^ by targeting certain elements in accountability with various data collection tools.

In line with our objectives, we aligned the case study with a developmental evaluation type of accompaniment,^35^ accompanying and supporting the development or adaptation of accountability tools co-constructed with the stakeholders, while taking into account their professional and management concerns.

The 4 cases chosen had to have been either initiated or accompanied by an HSSE. The choice of 4 IPPIs attested to a suitable sample in terms of representativeness and presented continuity with research carried out on their intervention previously, thus a sample by reasoned choice.

**Figure 1.**
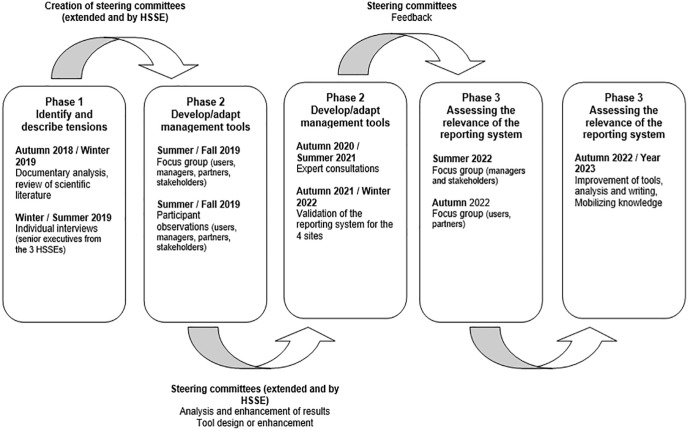
Phases and periods of research data collection.

The research project was carried out in 3 phases (Figure 1):

### Approaches to the co-construction and development of the communities

Our theoretical posture is based on the integration of practical and scholarly knowledge.^36^ Its epistemological scope is rooted in pragmatic approaches to co-construction and community development, which have helped define a participatory relationship to knowledge developed with stakeholders, managers, and community partners and users/citizens.

The approaches to the co-construction^37^ and development of the communities^38^ are part of the reference framework of this research.^39^ They promote the meaningful and influential participation of various actors in relation to the sharing of knowledge, experience and resources, which could improve the quality of services and allow individuals to participate in a more democratic decision-making process. As part of this research, the co-construction process takes shape through the involvement of the steering committees of each site and the extended steering committee, and through various co-construction activities carried out with territorial partners (community, municipal, police, education, etc.).

### Data collection

Data collection was conducted between 2019 and 2022. Three methods were used: (1) Documentary analysis of the literature produced by and about each of the IPPIs; (2) Participant observations on the accountability practices and on the conduct of team meetings between IPPI workers and managers; (3) Individual interviews and focus groups^2^ (Table 3).

**Table 3.** Data collection.

Documentation	Observations	Interviews	
Review of IPPI internal documentation	Observation grid for accountability elements	Semi-structured individual interviews	Focus group
	51 h	Executive (n = 12)	IPPI workers (n = 25)
			Managers (n = 12)
			Partners (n = 15)
			Citizens-users (n = 24)
		Total : 88 participants	

The data collection tools (interview guides and observation grid) were co-constructed with the steering committees during the first phase (Figure 1). Previous research on IPPIs^40,41^ has demonstrated the need to examine accountability in outreach interventions and to identify the mechanisms that hinder and those that facilitate the exercise of this responsibility. We therefore chose to use both individual and group semi-structured interviews, as they allow us to obtain relatively precise answers to questions focused on objectives, and to delve more deeply into the relatively limited topic of the research project, while allowing us to ask for clarifications. Here is an example of a question about governance asked during a semi-structured individual interview with an IPPI’s partner: “Can you give us examples where you feel like a partner with the HSSE and others where you don’t?”

Participants for the interviews and focus group were recruited internally: IPPI workers via a proposal from the manager to the researchers, then partners and citizen users were approached by the IPPI workers to find out if they were interested in participating in the research. The representativeness of the interviews and focus groups is strong because, in all cases, virtually all managers and stakeholders were involved in the project. Recruitment by purposive sampling also respected the criterion of relevance, particularly with partners and citizens. Each interview and focus group began with the explanation and signature of the consent form approved by the Ethics Committee. Interviews were recorded, with consent being sought from those involved on each occasion. Data collection was affected by the COVID-19 pandemic. Some interviews and focus groups took place in person, others virtually. They were conducted by the researchers responsible for each site, the project coordinator and research assistants. Interviews lasted between 1 h and 1 h 30 min.

Observations were carried out by the researchers responsible for each site, the project coordinator and research assistants. The observation notes were structured according to the type of activity and the number of people present at the time of the accountability data entry observations. Accountability data entry included institutional or in-house tools, their format (text, table, etc.), the concrete execution of the entry (technical obstacles, determination of content, etc.), the time dedicated, differences between the intervention carried out and what was entered, the context (presence(s), at what time, other tasks to be carried out simultaneously, etc.) and comments from the intervener. The research team also observed team meetings and conducted focus group interviews with users, partners, practitioners and managers on their perceptions of accountability.

### Analysis

The analyses were initially carried out by site, with instances of feedback on the ground, and then again from a comparative perspective. Our data analysis is primarily based on Paillé and Mucchielli’s^42^ descriptive thematic analysis approaches and Miles et al’s^43^ qualitative data analysis method. According to Paillé and Mucchielli, qualitative analysis typically involves a thematization process, which is a descriptive and exploratory framework that is contextual. Themes were identified through intra-site coding that was linked to research objectives, based on thematic analysis of documentation, individual interviews, participant observations, and group interviews conducted at each site. This process of collection was inscribed within a multiple case study design.^34^ Verbatim transcription of the interviews in NVivo 11 facilitated the codification and thematic grouping of topics discussed, while considering emerging themes.

After conducting a thematic analysis of the 4 IPPIs, we triangulated the data from various sources and compared selected themes to identify common and divergent issues with a comprehensive multisite view. Themes included issues, obstacles, tensions, type of governance, territory, IPPI mandate, issues, activities, and effects not considered in accountability, especially quantitative and qualitative data.^34,43^ The identification of common themes has led to the recognition of five dimensions essential in an accountability mechanism in IPPIs.

For each case, we produced a preliminary report and a summary report. The research team analyzed and discussed all ambiguous points from a perspective of co-construction integrated into data analysis and regular team discussions.

The data analysis, shared and co-constructed during the second phase (Figure 1), gave rise to some 20 research team meetings to produce an accountability framework comprising five dimensions, elements and accountability indicators. Based on this framework, each IPPI chose the relevant and feasible indicators for its own use, which varied according to the IPPI’s territory and particularities.

## Results

In this section, we present the internal analysis of each case, their initial conditions of emergence, the governance structure and the performance dimensions of the care and service trajectory. The Table 4 summarizes the characteristics of each case.

**Table 4.** Descriptions of the 4 cases.

	Case A	Case B	Case C	Case D
Type and number of personnel3	4 Workers 1 Clinical expert 1 Manager Partners	4 Workers 2 Specialized part-time workers 1 Manager Partners	4 Workers (including 2 part-time) 4 specialized part-time workers 1 Manager Partners	12 Workers 5 Managers Partners
Target population	Population of a high-density disadvantaged neighbourhood 10 000 pop.	Small town population 5700 pop.	Population of 2 disadvantaged neighborhoods 11 000 total pop.	Low-cost housing residents 500 pop.
Governance structure	Principal leadership of the HSSE (General Social Services Department) with partners in the community	Principal leadership of the HSSE (Public Health Department) with partners in the community	Principal leadership of the HSSE (General Services Department) with partners in the community	Shared leadership between partners (including HSSE)

### Case A, a high-density urban sector

#### Initial conditions and description

IPPI A is deployed since 1999 by the HSSE of the territory, following a study of the municipal district that revealed a high number of social issues and vulnerabilities (crime, social isolation, very recent immigration, poverty, low education levels, substandard housing, etc.) in a specific and very densely populated part of the territory. Several community organizations offer services in various areas in the territory. The IPPI, which is initially intersectoral (HSSE, community organizations, police, school and municipality), swiftly becomes overseen solely by the HSSE.

This IPPI is characterized by the stability of the team and the building of a relationship of trust with the populations. The team moves throughout the neighborhood and meets with residents and community organizations. They identify issues, as well as the resources available in the community and in the HSSE, and guide individuals toward these resources, while giving them the necessary tools to become independent.

#### Levers and tensions in terms of governance

With regard to structural levers, decision-makers within the HSSE recognize the IPPI for its positive effects on citizens-users and the community: “*We’ve already been commended by the managing director*” (IPPI worker). However, recognition of and support for the IPPI are related to the awareness and engagement among the current leadership: “*Either we believe in it, or we don’t*” (manager). And yet, competing institutional rationales persist, to the detriment of the philosophy of the IPPI: “*In our health sector, medical leaders are influential, which takes a big piece of the pie. I mean, that’s one of the elements at play; this is the health sector, after all*.” (manager).

Several successive organizational changes have also created a distance between the decision-makers and the ground. These notably include the reform of 2015, which brought about mergers that led to the establishment of imposing HSSE with over 10 000 employees: “*Previously, within the IPPI [. . .] the director is at the top on the* third *floor, and knows the team very well. Today, we have approximately 16 000 people, so how can 3 workers distinguish themselves?*” (IPPI worker).

Levers of knowledge, communications and resources are interlinked around the question of accountability. The issue is based on the knowledge and recognition of the added value of the IPPI through the quantitative measurement of its effects on the individuals receiving support, on the community and on the territory. Budgetary cuts have recently diminished the team and put the sustainability of this intervention at risk. The issues of the powers related to structural and resources levers are particularly apparent in this context.

However, in terms of the levers of connectivity—the very essence of the IPPI—there is strong support from partners (see bellow). However, being mainly made up of community organizations, they have very little influence within the HSSE.

#### Performance of the pathway

One of the strengths of the IPPI lies in its direct accessibility for workers within the environment and their adjustments to the population. The citizens-users underscored this human quality, which is conducive to maintaining relationships and building self-esteem: “*they don’t treat people like numbers, which is what doctors do. Instead, they try to empathize with how we feel*” (citizen-user).

In terms of the intersectoral partners, the organizations recognize the role of “connectivity”^32^ played by the IPPI in the HSSE and its territorial network. The IPPI workers can directly guide the citizens-users toward services or be called upon by partners to create common strategies with a view to reaching out directly to a specific clientele.

As part of a philosophy of equity, the IPPI aims to foster better access to the health system among a “distrustful” clientele that has several vulnerabilities. However, their efforts are restricted by a lack of continuity and coordination between the sectoral and intersectoral services of the HSSE and its territorial network, which leads to a bottleneck in the system.

Lastly, the lack of strategic and tactical support to the team leads to the significant weakening of the IPPI, which leads to a high risk of an end to the IPPI services.

### Case B, a town in a rural context

#### Initial conditions and description

IPPI B is located in a small town. Following the occurrence of a serious accident, a temporary recovery team was established during and after this accident. It was made up of emergency psychosocial workers, with a focus on individual health and security. Three years later, under the auspices of the directorate for public health (DPH), a permanent IPPI team was created with the mandate of working toward prevention and community and individual resilience.

#### Levers and tensions in terms of governance

The identified tensions mostly stem from governance at the tactical level. The DPH has implemented and directed the IPPI. The expertise of a manager at the strategic level has enabled guidance and support for the IPPI at the operational level. But the absence of a manager at the tactical level generates dysfunctions. For the DPH

[*. . .*] *the link with the clinical management bodies is what I find difficult. A clinical management body that is in charge of staff [. . .] if I was a head of department, I would have statutory staff with them just to keep abreast of things, to be aware of my teams [. . .]. But that’s where there’s a gap [. . .] I don’t consider myself to be a head of department* (DPH).

The issue becomes tangible in terms of impairing fluidity in communications, through fragmented knowledge-sharing concerning the IPPI, compromising the management of teams, coordination and communications between the IPPI and the clinical management bodies of the HSSE. The strategic manager gave the impression that assigning a tactical manager to the team with a view to replacing them at the clinical level could put the intervention at risk. This situation involves several elements linked to their role, knowledge of the IPPI, expertise in the development of communities, but could also improve links between the management bodies. We therefore take note of a recognition of the IPPI at the strategic level, even though significant gaps in awareness remain. Nonetheless, the levers of knowledge, communications and connectivity are clear among the intersectoral partners and citizens-users (see below).

The question of resources is linked to a knowledge of the IPPI, but it is not currently a source of tension. However, it remains a concern for the workers:

*I think that we will need to continue to convey the value of [the IPPI]. Because they involve very administrative rationales [. . .], if there are cuts to be made [. . .] it’s the type of team that it would be easy to have removed* (IPPI worker).

#### Performance of the pathway

The contribution of the IPPI as a link in the community-integrated pathway was mentioned by the partners and the citizens-users: the IPPI is relevant and plays a significant role in the community. The partners mentioned that the IPPI recognizes their contribution and enriches their interventions thanks to the support provided. According to one partner:

*I find that it creates a genuine continuum. [. . .] I will do something [with the citizen] and the next person [the IPPI worker] will take over. That’s what has been created, and it’s not something that was normal in [the HSSE]* (partner).

The IPPI facilitates access to the HSSE while also involving cumbersome administrative procedures that can make the implementation of certain projects more difficult.

We have noted that the IPPI also increases citizen participation. The workers are highly meaningful in the eyes of the citizens-users, who gave details of accessibility to services, the relational proximity and the adjustments to the needs of individuals and the community, notably in terms of prevention. The IPPI has changed the vision of the community, developed a caring community and fostered a sense of integration, while working with partners on projects from within the community while referring citizens to them on a regular basis. The IPPI offers additional and different services from those provided by the HSSE, such as assistance with housing or employment, working toward equity in terms of the needs of citizen-users in vulnerable situations.

### Case C, neighborhoods in an urban environment

#### Initial conditions and description

The IPPI was rolled out in 2009 in 2 different neighborhoods within a town, with a view to reaching a population living in a context of poverty and social exclusion, which was not making use of local services. In 2016, each area had approximately 5000 inhabitants, 13% of whom were immigrants for one, and 21% for the other.^44^ The renting of an apartment in each of these neighborhoods allows the IPPI to be close to the community. The directorate for general services is responsible for the IPPI, which is made up of 4 social workers equally distributed in the neighborhoods. These workers undertake various tasks (psychosocial welcome, group and individual interventions, consultation with partners, social pediatrics, etc.). Since 2014, actors from 4 different programs in the HSSE have been working to support the IPPI.

#### Levers and tensions in terms of governance

The IPPI was created with the support of upper management. Nonetheless, the new managers do not know the IPPI well, nor its impact on the relevant neighborhoods. This lack of understanding makes the IPPI more fragile, and put it at particular risk in 2015. “*No money, no services. It’s necessary to prioritize*” (executive). An understanding of this “*range of services* that is *specific, different, and neither widespread nor widely known*” (executive) is essential. There is a lack of leadership that would be needed to increase knowledge among the departments involved and workers in other programs.

The standardized accountability procedures of the health care network is not compatible with the IPPI and do not reflect the reality of what has been accomplished. At present, everything depends on demonstrating the potential long-term impacts. The absence or lack of knowledge at the decision-making levels weakens the IPPI. Certain resources can be cut for the benefit of other priorities.

The IPPI has developed a connectivity with its partners through the establishment of strong links and fluid communications where their expertise is recognized and becomes complementary.

#### Performance of the pathways

The citizen-users who were interviewed appreciate the IPPI, as it is different from what they have experienced within the health and social services network. One citizen-user observed that:

*In hospital, you’re given a number, but when you arrive here, they call you by your name, you are given a proper welcome [. . .] there’s no timer running down. They resolve your problem and afterwards, they turn to other matters. [. . .] The IPPI worker is my confidant* (citizen-user).

The accessibility of a service is fostered through its integration within the neighborhood and by building trust. The IPPI allows the HSSE to fulfill its population responsibility in an equal manner, including to citizens that it is unable to reach through its standard facilities. The IPPI provides information on the needs of the population and allows the HSSE “*to adapt [its] range of services*” (executive).

The IPPI operates using integrated services offered by the HSSE and through an intersectoral approach with its partners. The IPPI is a key element in the continuity and fluidity of services. The managers consulted perceive their ability to create effective service pathways.

*Our [HSSE] must recognize [the IPPI]. That is how we will be able to create fluid pathways, how we will be able to create service continuums, how we will empower people because we will be closer to them and able to better support them* (manager).

These community-integrated service pathways have the potential to increase their performance if the funding is granted, as a result of recognition by the decision-makers.

### Case D, a micro-territory in an urban context

#### Initial conditions and description

Built in the 1970s and located between 2 neighborhoods characterized by a high level of material and social disadvantage, Case D is a low-income housing complex managed by the municipal office for housing, which since 2019 has brought together over 400 residents of different nationalities spread across over 70 places of residence. In 2007, actors from several bodies (municipal office for housing, community organization for families, youth center, various municipal housing services, HSSE, municipal police service) pool their expertise and resources and agree to work in partnership to support the living environment and to tackle various emerging issues such as vandalism, drug use, and integration challenges for immigrant residents. This is how the IPPI D came to be established.

#### Levers and tensions in terms of governance

Regarding the structure, the IPPI is based on the shared partnership and leadership of the different bodies involved based on the recognition that the IPPI belongs to all of the partners and, beyond their own mission, the common mission of the IPPI is to improve the health and well-being of the residents. The IPPI is coordinated in a joint manner by the intersectoral partners that meet within various committees (committee of managers, roundtable bringing together managers, workers and residents, roundtable of IPPI workers) to make decisions and to provide collective guidance. Each of the actors involved takes on clearly defined and agreed-upon roles. Regarding the resources, each of the organizations contributes in terms of human and material resources (premises, equipment) to support the IPPI. The nature of the collaboration and the actual activities of the team are specificities set out by the executives, the managers and the workers, allowing the workers to have rapid access to the different partners, as well as information exchange between them. The frequency of the meetings of the different committees is conducive to establishing ties between the actors involved and reflexivity.

*I find that through the partnership, I don’t feel alone. [. . .] When there’s a problem, I don’t feel that I’m all alone. I share it with others, and it’s as if I’m not responsible for the others either; we make decisions together, we put our heads together to find the best strategy. I think that working together is a strength of ours* (IPPI worker).

Over the years, recognition of the genuinely shared ownership of the IPPI has been a source of discussion. In addition, departing from one’s mission and usual field, while also respecting the responsibilities and remits of the other actors involved is one of the challenges faced. Lastly, the lack of financial and human resources remains a concern with regard to the major needs encountered, but this does not call the relevance of the IPPI into question.

#### Performance of the pathways

The main benefits of the IPPI mentioned by all the actors involved are the proximity and accessibility between the tenants and the workers. In addition, the tenants consider that the IPPI has allowed them to become more aware of the services available, to better integrate and adapt to their environment, to become more involved in various activities and committees. Lastly, they observe a reduction in crime and have a sense of well-being and do not wish to leave their environment. Moreover, the partners involved consider that the decompartmentalization and the expansion of their network has enabled various obstacles to be eliminated, allowing for enhanced and more timely interventions.

*When help is needed, well, we can always count on someone. And we can talk to anyone, at any time. They will always be there* (tenant).

*The other advantage, well, as I was on the streets for a long time, I had huge problems with structural barriers. When someone needs help, you’re faced with schedules, with whatever issue [. . .]. It’s one of the advantages of the community-integrated interventions. In relation to the individuals from within the environment, it’s definitely more accessible, and at the same time more personalized* (IPPI worker).

The Table 5 summarizes the action levers and performance dimensions for each case.

**Table 5.** Summary of action levers and performance dimensions by case.

		Case A	Case B	Case C	Case D
Action levers	Structure	-	+/-	-	+
	Resources	-	+/-	-	+
	Communication	+/-	-	+/-	+
	Connectivity	+	+	+	+
	Knowledge	+/-	+/-	+/-	+
Performance	Accessibility of care and services	+	+	+	+
	Adjustment to the needs of the population	+	+	+	+
	Coordination and continuity of care and services	+/-	+	+/-	+
	Equity	+	+	+	+

“-”: Absence/missing/issues, “+”: Presence/strength, “+/-”: Mixed assessment.

## Discussion

In the presentation of the 4 IPPIs, some common points stand out, notably the innovative nature of these IPPIs within their environments. They were implemented in response to complex social issues noted by several actors associated with the different territories and which required alternative responses to the conventional routes (initial conditions of implementation). Their emergence therefore led the actors concerned—from both institutional and community environments—to adopt different ways of working. Additionally, innovative working methods showed success with noticeable advantages for the specific communities targeted. For each IPPI, relations with the partners are solid and constructive, and the impacts are positive in terms of accessibility, adjustments, citizen participation and equity for the citizen-users. In this regard, the 4 IPPIs contribute to establishing performing community-integrated pathways, according to the selected definition of performance.^14^

Nonetheless, and beyond the contextual differences (rural/urban, the driving element or even the political context), a major difference between the sites concerns the actors responsible for implementing the initiatives: IPPIs A, B, and C report directly to the HSSEs, while IPPI D is the responsibility of a group of institutional and community actors.

This initial distinction generates 2 differing modes of governance, which themselves have different ramifications for the IPPIs and the performance of the pathways. IPPIs A, B, and C remain associated with their respective HSSEs and therefore integrate themselves into a hierarchical governance. While this situation gives legitimacy and institutional support to the IPPIs, we have also noted significant governance issues linked to the structure (variable recognition according to the actors, distance and gap between the values conveyed by each level of governance, variable strength of special interest and lobbying groups), to the resources (lack of financial, material and human resources), to knowledge and communications (lack of transfer of knowledge about the IPPI). Although these issues may not directly affect the impacts on the ground for citizens-users, they lead to questioning the credibility of the IPPIs and endanger them within their establishments. Notably, this occurs through a lack of data on the benefits, which are too focused on quantitative indicators and underestimate the real benefits and the performance of the service pathways and therefore of the IPPIs themselves.

Nonetheless, these 3 IPPIs do not form a homogeneous group. The IPPIs are not affiliated to the same department (General Social Services, Public Health and General Services) within the HSSEs. Certain issues are specific to each of them, notably the distance between the strategic and operational values in one case, an absence at the tactical level in another case, and different levels of recognition according to the leadership of the projects (and of their position in the governance structure). However, these tensions weaken the ties between governance levels needed to ensure the credibility and maintenance of the IPPI within establishments and territories.

IPPI D belongs to a group of intersectoral actors (including the HSSE) and is based on shared and collaborative multilevel governance. This certainly constitutes a challenge, as it requires a significant level of investment from all the partners and involves emerging tensions related to the distribution and limitations of each of their roles; challenge that has been documented in other contexts.^45^ However, in comparison with the other cases, the relevance of IPPI D is not called into question. The actors at all the levels of governance know and meet with one another, share information, are aware of the benefits and issues related to the IPPI, make adjustments, agree upon a common purpose beyond their initial missions, have a common understanding of their roles and responsibilities, and are willing to defend this intervention.

These 2 modes of governance influence the perspectives on these IPPIs, despite the incontestable benefits for the citizens-users targeted within each site. The future of case D is not threatened, while those of the 3 others are weakened (to varying degrees). Their survival depends to a significant extent on the support of the upper management of their affiliated HSSE, in a context where there is a notable employee turnover.

We are faced with a contradiction: there seems to be a consensus within the health and social services network and a desire on the part of the population for local services. The IPPI is particularly valued at the strategic level. This is true not only within HSSEs, but also at ministerial level. Integrated place-based interventions are at the heart of recent ministerial recommendations,^46^ and even more recently a reference framework entitled “Improving access, quality and continuity of integrated place-based services” has been produced by the Health and Social Services Ministry and published this year.^47^ But there is a discrepancy with the concrete means provided to make these initiatives work at the operational level. And our findings seem to suggest that one of the cogs in this contradiction is the distance between levels of governance, in terms of collaboration and shared values.

As an example of this paradox, in one of the HSSE to which one of the IPPIs is affiliated, new community-based team projects are being developed without considering what has been done within the IPPI, and what is therefore being dismantled.^5^

These contradictions also resonate with the findings of a scoping review of cross-sectoral and multi-organizational interventions^15^: while there is a consensus that these interventions need to work in this way to address the complex challenges of homelessness (in this context), few studies have examined the governance issues implied by intersectorality and multi-organizationalism. The studies reviewed pay little attention to the dynamics between governance actors, or to how actors from different sectors and at different levels interact with each other and how they evolve together. This scoping review raises the question of whether, more broadly, the actors who create policies, strategies and structures to address homelessness do not approach governance in a strategic and planned way.

Finally, these findings shed light on our initial question about the link between IPPI governance and pathway performance. The initial conditions (notably the actor or group of actors responsible for the initiative) and the levers of actions (notably related to structure, resources and communications) that favor a *collaborative* multilevel governance of the IPPIs are particularly crucial to determine and support the *sustainability* of the benefits of the community-integrated pathways, in terms of accessibility, adjustments and equity. Even in a mode of hierarchical governance such as that which underpins the HSSE (for IPPIs A, B, and C), the operational level can have a positive impact on communities, particularly through collaborations with the intersectoral partners, as long as they receive strategic support (which is essential for their survival). However, the study of IPPI D suggests that a collaborative and shared multilevel governance would contribute to the longevity of the actions and benefits of the IPPI and would avoid any questioning of their existence and relevance.

### Limits

Our study has limitations, including the fact that only one case (on 4) is exhibiting collaborative multilevel governance. Our analysis of the relationships between governance mode and pathway performance would have been strengthened by additional cases of shared responsibility.

Other limitations include the time constraints on managers and stakeholders to participate in the research project. As a result, we were not able to interview all stakeholders or at all the stages we had planned (in particular to test the tool we had developed). In addition, the COVID-19 pandemic limited our access to the field: no research could be conducted in the HSSEs for several months. This caused a rupture in the field and hindered the reconnection with partners and users upon our return. Many people had departed, and our contacts were no longer current.

## Conclusion

In this article, we have highlighted the initial conditions for the implementation of 4 IPPIs, as well as the actions levers and tensions in the governance in which they are embedded. The levels of performance of the community-integrated pathways have also been explored in terms of accessibility, adjustment to the needs of the citizens-users and equity.

One might assume a priori that an intervention that is the responsibility of one sole institution would be easier to maintain than one that is the responsibility of multiple actors with different missions and approaches. And yet, our results show that this is not necessarily the case. Even if an intervention falls under the responsibility of one sole institution, the levels of a multilevel governance must be close, interact, share knowledge and discuss issues, notably in the context of health and social services organizations where employee turnover is high. In addition, if the study of IPPI D with collaborative intersectoral governance appears to be the most promising, it is important to remain mindful that this approach is highly demanding, as it requires a strong belief in the need to innovate and work in an intersectoral manner, an ability to change working methods and depart from the usual mandates, as well as the implementation of equal relationships between the partners. However, IPPI D shows that this route is both possible and advantageous.

These findings reinforce the notion that intersectoral initiatives aimed at improving access for the most vulnerable or marginalized individuals in the healthcare and social services network necessitate multilevel, collaborative governance to bolster the process of adapting the organization of healthcare and social services to promote greater equity.
